# Bone Metabolism and Dental Implant Insertion as a Correlation Affecting on Marginal Bone Remodeling: Texture Analysis and the New Corticalization Index, Predictor of Marginal Bone Loss—3 Months of Follow-Up

**DOI:** 10.3390/jcm13113212

**Published:** 2024-05-30

**Authors:** Tomasz Wach, Piotr Szymor, Grzegorz Trybek, Maciej Sikora, Adam Michcik, Marcin Kozakiewicz

**Affiliations:** 1 Department of Maxillofacial Surgery, Medical University of Lodz, 113 Żeromskiego Str., 90-549 Lodz, Poland; piotr.szymor@umed.lodz.pl (P.S.); marcin.kozakiewicz@umed.lodz.pl (M.K.); 2 Department of Oral Surgery, Pomeranian Medical University in Szczecin, 70-111 Szczecin, Poland; g.trybek@gmail.com; 3 4th Military Clinical Hospital in Wroclaw, ul. Rudolfa Weigla 5, 50-981 Wroclaw, Poland; 4 Department of Maxillofacial Surgery, Hospital of the Ministry of Interior, Wojska Polskiego 51, 25-375 Kielce, Poland; sikora-maciej@wp.pl; 5 Department of Biochemistry and Medical Chemistry, Pomeranian Medical University, Powstańców Wielkopolskich 72, 70-111 Szczecin, Poland; 6 Department of Maxillofacial Surgery, Medical University of Gdansk, 80-210 Gdańsk, Poland; adammichcik@gumed.edu.pl

**Keywords:** BMI, bone metabolism, calcium in serum, corticalization phenomenon, dental implant lost, general condition, HDL intraoral radiographs, LDL, marginal bone loss, phosphates in serum, PTH, radiomics, texture analysis, TSH

## Abstract

**Background/Objectives:** The general condition of implantology patients is crucial when considering the long- and short-term survival of dental implants. The aim of the research was to evaluate the correlation between the new corticalization index (CI) and patients’ condition, and its impact on marginal bone loss (MBL) leading to implant failure, using only radiographic (RTG) images on a pixel level. **Method:** Bone near the dental implant neck was examined, and texture features were analyzed. Statistical analysis includes analysis of simple regression where the correlation coefficient (CC) and R2 were calculated. Detected relationships were assumed to be statistically significant when *p* < 0.05. Statgraphics Centurion version 18.1.12 (Stat Point Technologies, Warrenton, VA, USA) was used to conduct the statistical analyses. **Results:** The research revealed a correlation between MBL after 3 months and BMI, PTH, TSH, Ca^2+^ level in blood serum, phosphates in blood serum, and vitamin D. A correlation was also observed between CI and PTH, Ca^2+^ level in blood serum, vitamin D, LDL, HDL, and triglycerides on the day of surgery. After 3 months of the observation period, CI was correlated with PTH, TSH, Ca^2+^ level in blood serum, and triglycerides. **Conclusion:** The results of the research confirm that the general condition of patients corresponds with CI and MBL. A patient’s general condition has an impact on bone metabolism around dental implants. Implant insertion should be considered if the general condition of the patient is not stable. However, CI has not yet been fully investigated. Further studies are necessary to check and categorize the impact of corticalization on marginal bone loss near dental implants.

## 1. Introduction

Marginal bone loss occurs when bone tissue resorbs near the neck of dental implants. Various factors can influence bone metabolism near the implant neck, such as cigarettes, oxidative stress, smoking, prosthetic restoration, surgical techniques, operator skill, torque during implant insertion, and the general condition (GC) of patients, including calcium phosphate metabolism and bone-healing capacity [1,2,3,4,5,6,7,8,9,10,11].

Some papers present immune incompetency as a low risk factor of dental implants failure. General health problems, e.g., osteoporosis and sometimes related with this, impact of bisphosphonate drugs on success rate of implants. All of the results were confirmed after the clinical problems [12,13,14]. Is there any possibility of detecting problems with bone before implantation, taking into account only radiological images?

The factors affecting bone metabolism include Body Mass Index (BMI), parathormone (PTH) level, thyroid-stimulating hormone (TSH) level, total levels of Ca^2+^ and phosphates in blood serum, total level of vitamin D3, levels of high-density lipoprotein (HDL) and low-density lipoprotein (LDL), and triglyceride levels in blood serum.

Body Mass Index (BMI) is a measure of body fat based on height and weight, defining conditions such as obesity when BMI exceeds 30 kg/m^2^. Higher body mass can positively impact mechanical loading and bone formation but also negatively affect bone metabolism primarily through decreased osteoblast differentiation and bone formation [15,16,17]. 

Parathyroid hormone (PTH)—84-amino acid peptide hormone—is crucial in calcium homeostasis, and it is synthesized in the chief cells of the parathyroid glands. PTH regulates calcium and phosphorus levels in serum, influencing bones, kidneys, and the gastrointestinal system. Decreasing levels of ionized calcium concentration in blood is a signal of PTH production and secretion; in contrast, when the level of calcium is increasing in serum, the production and secretion of PTH is limited. Depending on its serum level, PTH can have either a catabolic or anabolic effect on bone metabolism, directly affecting osteoblasts and osteocytes and indirectly influencing osteoclasts [18,19,20]. 

Thyroid-stimulating hormone (TSH) regulates thyroid hormones essential for bone metabolism. Both hyper- and hypothyroidism negatively impact bone tissue, with hypothyroidism leading to fatigue and obesity, while hyperthyroidism increases the risk of cardiovascular diseases and osteoporosis. TSH directly affects bone resorption and activates osteogenesis [21,22,23,24,25]. 

Phosphates also impact bone metabolism. Hyperphosphatemia decreases calcium efflux from bone, while hypophosphatemia has the opposite effect. Phosphates in the diet increase PTH levels in serum, maintaining normal calcium and phosphorus levels. Calcitriol enhances calcium and phosphate absorption in renal physiology, but excessive levels can disrupt bone metabolism [26]. 

Cholesterol, classified based on density as low-density lipoprotein or (LDL) as “bad” cholesterol and high-density lipoprotein (HDL) or “good” cholesterol, affects bone metabolism by influencing bone resorption and reducing bone formation, such as by inhibiting osteoblast differentiation [27]. 

Until now, the corticalization index was not considered in the literature. We were the first to calculate, evaluate and characterize this index. This phenomenon, evaluated on a pixel level, may be the first predictor of marginal bone loss which leads to implant failure.

The aim of this study was to present, analyze, and evaluate the correlation between the new bone index (Corticalization) and general health condition that could indicate marginal bone loss leading to dental implant failure.

## 2. Materials and Methods

Presented research was based on 2196 retrospective radiological data of oral implantological surgery treatment.

### 2.1. Inclusion Criteria

Patients were included into the study taking into account few inclusion criteria. Inclusion criteria were integrated implants after 3 months, 18 years old, bleeding on probing <20%, good oral hygiene, gingival pocket depth 3 mm or less, and two-dimensional radiographs taken during routine checks and regular follow-ups. Blood tests took into account ion and hormone levels: TSH (normal range 0.23–4.0 µU/mL), PTH (normal range 10 to 60 pg/mL), glycated hemoglobin (normal range < 5%), ions Ca^2+^ (normal range 9–11 mg/dL), and vitamin D3 (normal range 31–50 ng/mL). The spin densitometry was also checked (Table 1). 

### 2.2. Exclusion Criteria

Patients were excluded from the study when there was an absence/low quality or lack of RTG images during the observation period, they lost the implant before 3 months of healing, they did not take laboratory tests, RTG images were defected during visual assessment, internal comorbidities were not well controlled, there were other immunodeficiencies, there was radiotherapy in their medical history, soft or/and bone augmentation, or there were cytostatic drugs in the medical history (Table 2).

### 2.3. Treatment Procedure

Dental implant insertion was performed by the same surgeon (M.K.) taking into account all protocols and guidelines. Three months after the healing period, implants were uncovered under local anesthesia and standard healing screws were inserted. The follow-up lasted for 3 months.

### 2.4. Data Acquisition

Data acquisition is detailed as described in the publication from the series in the following MDPI journal [28]: 

## 3. Results

**Figure 1:**
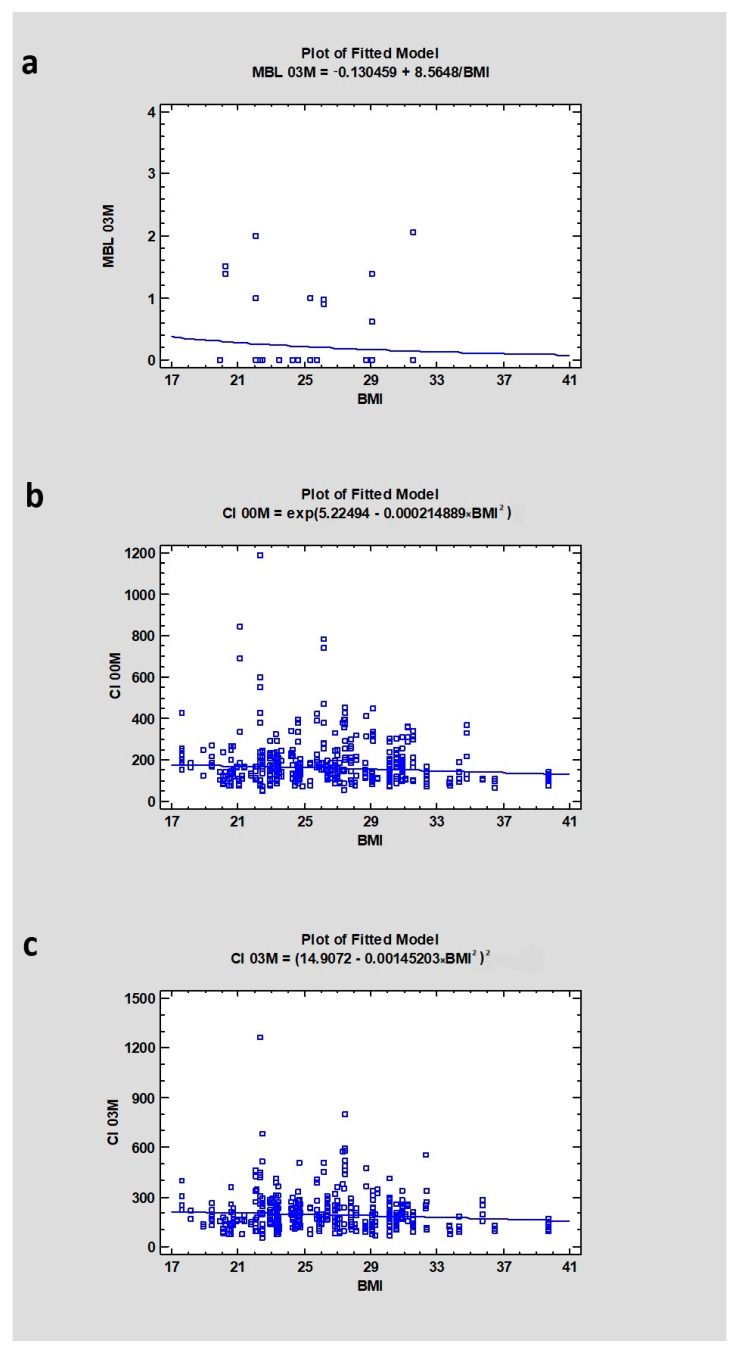
Dependencies for all samples: (**a**) Dependency of Body Mass Index on marginal bone loss after 3 months of healing. (**b**) Dependency of Body Mass Index on Corticalization Index in the initial period. (**c**) Dependency of Body Mass Index on Corticalization Index after 3 months of healing. Abbreviations: MBL03—marginal bone loss after 3 months of healing; CI00M—Corticalization Index in the initial period; CI03M—Corticalization Index after 3 months of healing.

The study revealed that the median value for BMI was 26.05 ± 4.26, and the marginal bone loss (MBL) after 3 months was 0.00 ± 0.78 mm, indicating a weak relationship between MBL after 3 months of healing and patients’ BMI (Correlation Coefficient, CC = 0.087; *p* = 0.49). The median Corticalization Index on the day of surgery (CI00M) was 146, which increased to 171 after 3 months (CI03M) (*p* < 0.001). There was a relatively weak correlation between CI00M and BMI (Correlation Coefficient, CC = −0.11) and also between CI03M and BMI (CC = −0.010), where *p* < 0.05, signifying statistical significance (Table 3 and Table 4) (Figure 1).

**Figure 2:**
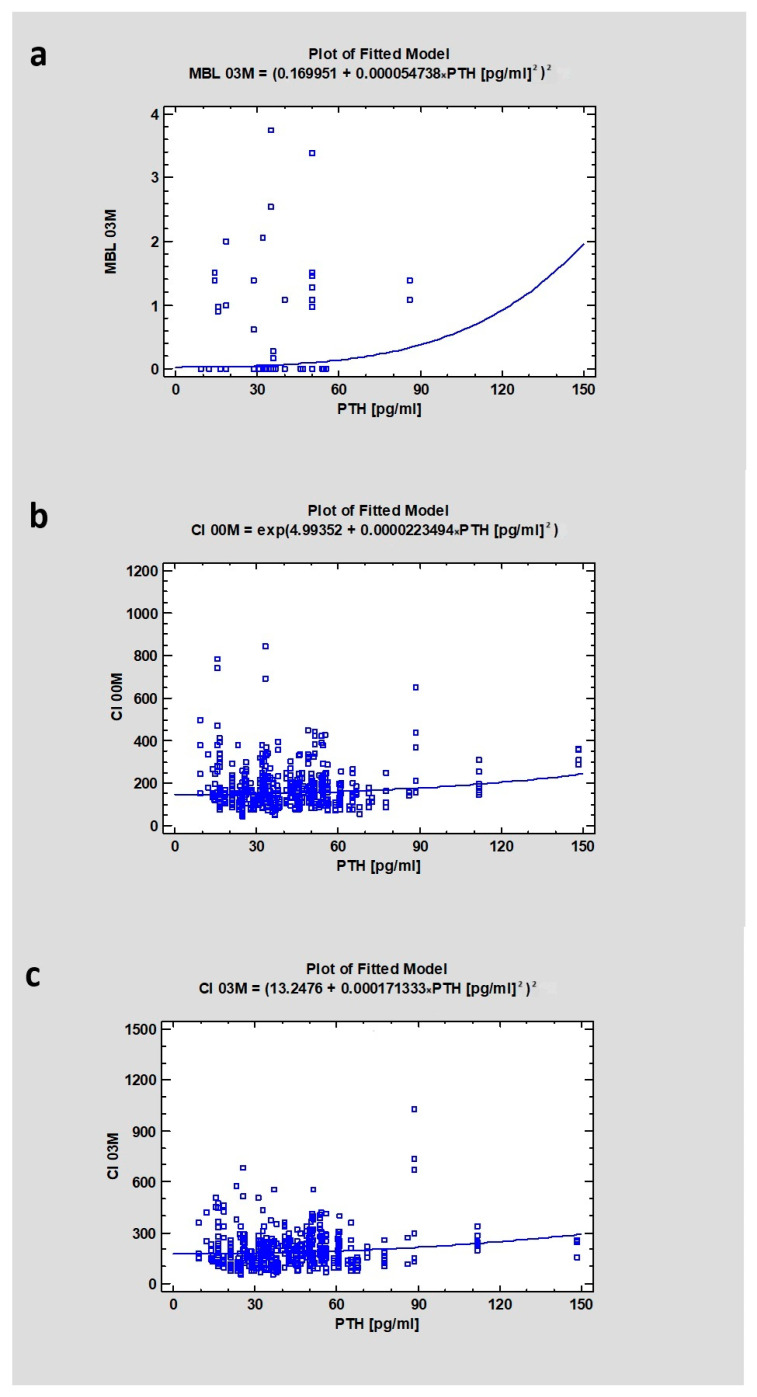
Dependencies for all samples: (**a**) Dependency of parathormone on marginal bone loss after 3 months of healing. (**b**) Dependency of parathormone on Corticalization Index in the initial period. (**c**) Dependency of parathormone on Corticalization Index after 3 months of healing. Abbreviations: MBL03—marginal bone loss after 3 months of healing; CI00M—Corticalization Index in the initial period; CI03M—Corticalization Index after 3 months of healing.

The median value for PTH was 38, showing a weak correlation with MBL (*p* = 0.18 for the 3-month observation period and CC = 0.14). However, the study revealed a weak relationship between PTH and CI00M (CC = 0.12) and CI03M (CC = 0.13), where *p* < 0.001 in both cases (Table 3 and Table 4) (Figure 2).

**Figure 3:**
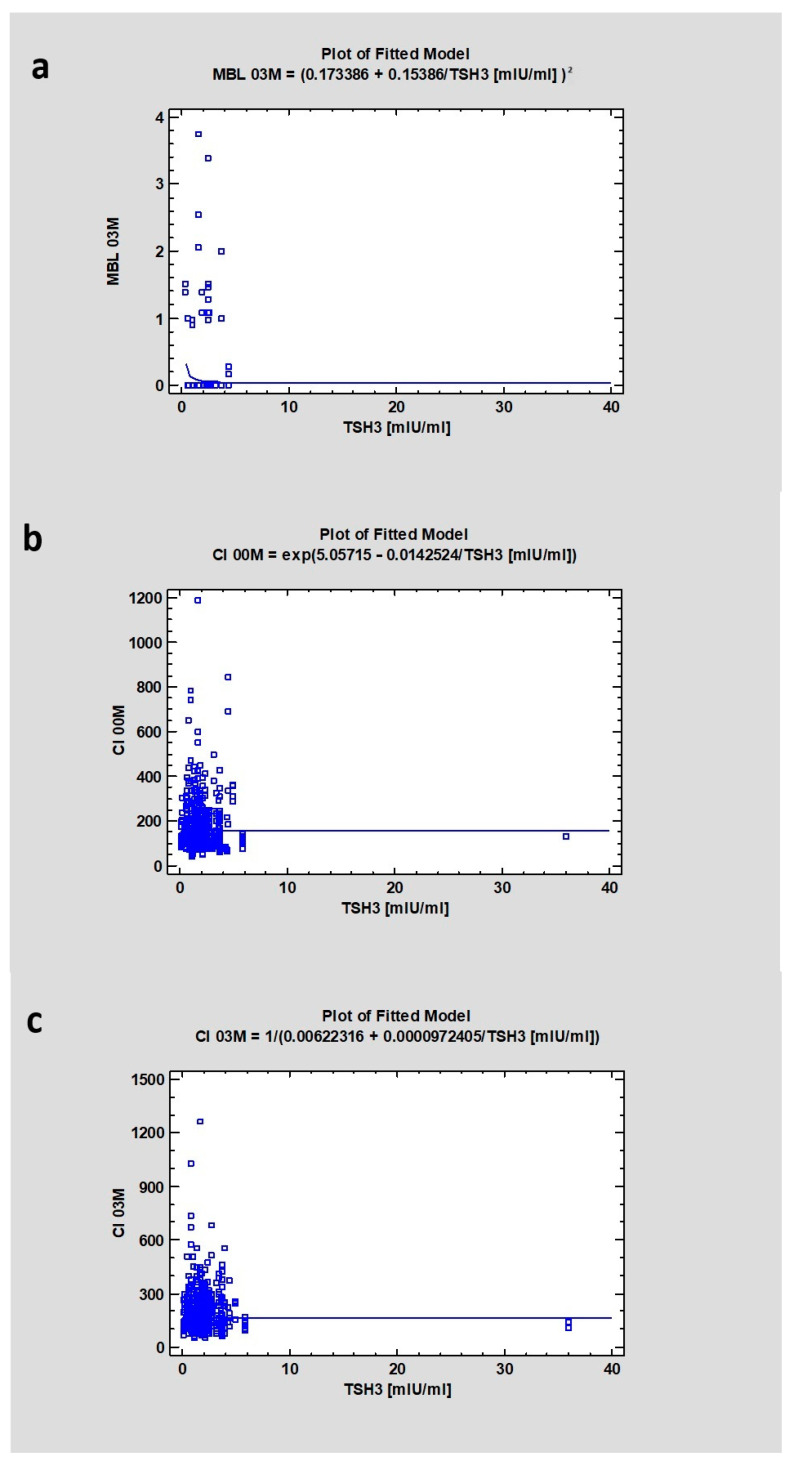
Dependencies for all samples: (**a**) Dependency of thyroid-stimulating hormone on marginal bone loss after 3 months of healing. (**b**) Dependency of thyroid-stimulating hormone on Corticalization Index in the initial period. (**c**) Dependency of thyroid-stimulating hormone on Corticalization Index after 3 months of healing. Abbreviations: MBL03—marginal bone loss after 3 months of healing; CI00M—Corticalization Index in the initial period; CI03M—Corticalization Index after 3 months of healing.

The median level of TSH was 1.56, and a weak relationship was found between TSH and MBL03M (CC = 0.16; *p* = 0.13). There was also a correlation indicated between CI00M (CC = −0.085, *p* < 0.05) and CI03M (again, CC = 0.1, *p* < 0.05) and the TSH level (Table 3 and Table 4) (Figure 3).

**Figure 4:**
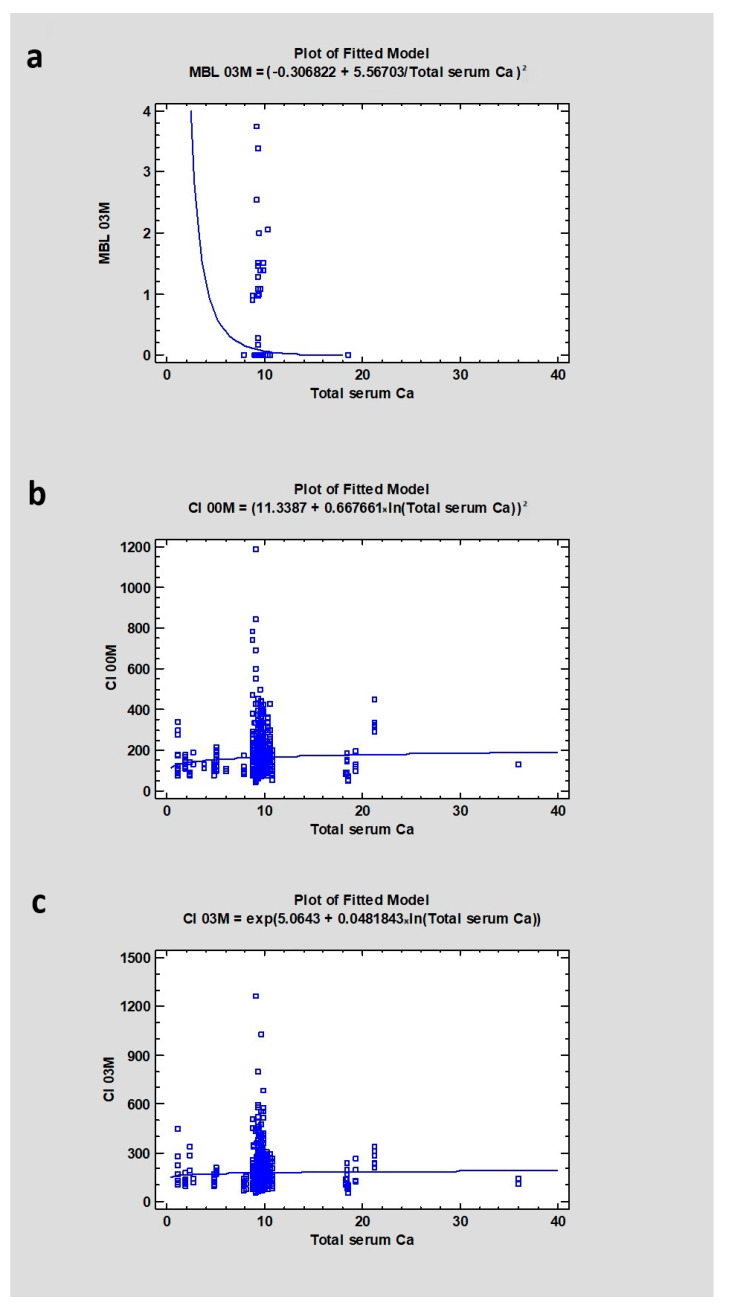
Dependencies for all samples: (**a**) Dependency of calcium level in blood serum on marginal bone loss after 3 months of healing. (**b**) Dependency of calcium level in blood serum on Corticalization Index in the initial period. (**c**) Dependency of calcium level in blood serum on Corticalization Index after 3 months of healing. Abbreviations: MBL03—marginal bone loss after 3 months of healing; CI00M—Corticalization Index in the initial period; CI03M—Corticalization Index after 3 months of healing.

A different correlation was indicated in the case of calcium in serum. The median for calcium in serum was 9.5. The research showed a relation between calcium level and CI00M (*p*-value lower than 0.05—statistically significant, CC = 0.1) and a weak relation between calcium and MBL (CC = 0.13, *p* = 0.23) and CI after 3 months of healing (CC = 0.044), where *p* > 0.05, indicating no statistical significance (Table 3 and Table 4) (Figure 4).

**Figure 5:**
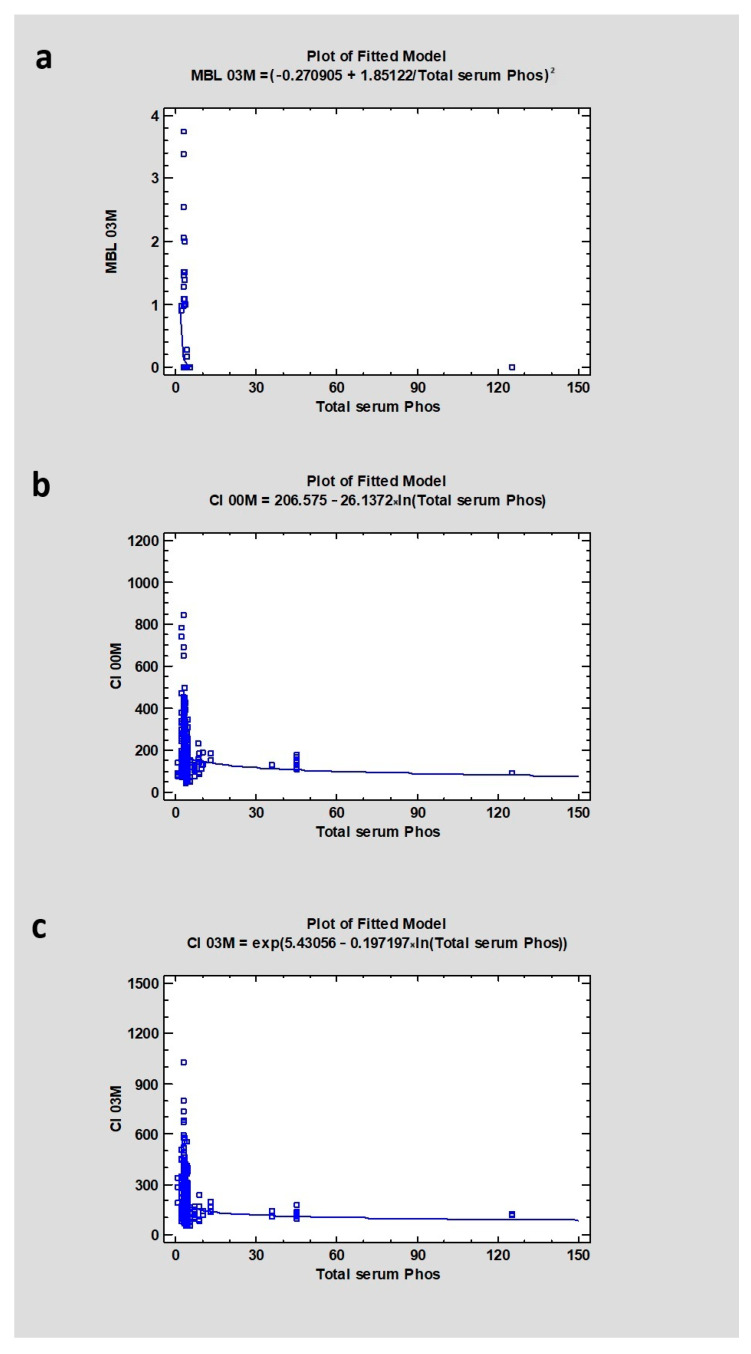
Dependencies for all samples: (**a**) Dependency of phosphates level in blood serum on marginal bone loss after 3 months of healing. (**b**) Dependency of phosphates level in blood serum on Corticalization Index in the initial period. (**c**) Dependency of phosphates level in blood serum on Corticalization Index after 3 months of healing. Abbreviations: MBL03—marginal bone loss after 3 months of healing; CI00M—Corticalization Index in the initial period; CI03M—Corticalization Index after 3 months of healing.

Regarding phosphates in serum, where the median was 3.3 ± 7.9, the study showed dependence with MBL03M (CC = 0.24), CI00M (CC = −0.14), and CI03M (CC = −0.23) with a *p* < 0.05, which was statistically significant (Table 3 and Table 4) (Figure 5).

**Figure 6:**
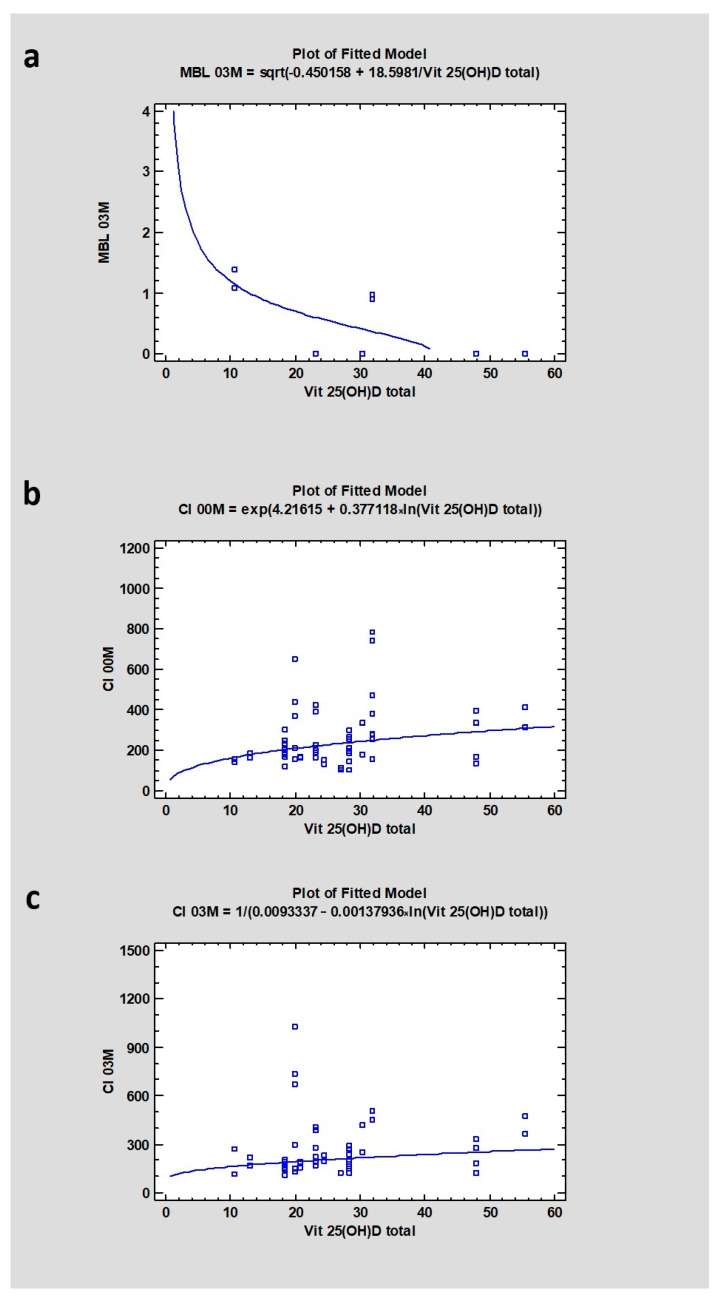
Dependencies for all samples: (**a**) Dependency of vitamin D level in blood serum on marginal bone loss after 3 months of healing. (**b**) Dependency of vitamin D level in blood serum on Corticalization Index in the initial period. (**c**) Dependency of vitamin D level in blood serum on Corticalization Index after 3 months of healing. Abbreviations: MBL03—marginal bone loss after 3 months of healing; CI00M—Corticalization Index in the initial period; CI03M—Corticalization Index after 3 months of healing.

The mean value for Vitamin 25(OH)D total in serum was 23.75 ± 13.55. The research revealed a strong correlation (CC = 0.75) with MBL after 3 months (*p* < 0.001), with CI on the day of surgery (CC = 0.29), with a *p* < 0.05, which was statistically significant. There was also a weak correlation between vitamin 25(OH)D and CI03M, where the p value was higher than 0.05 (Table 3 and Table 4) (Figure 6).

**Figure 7:**
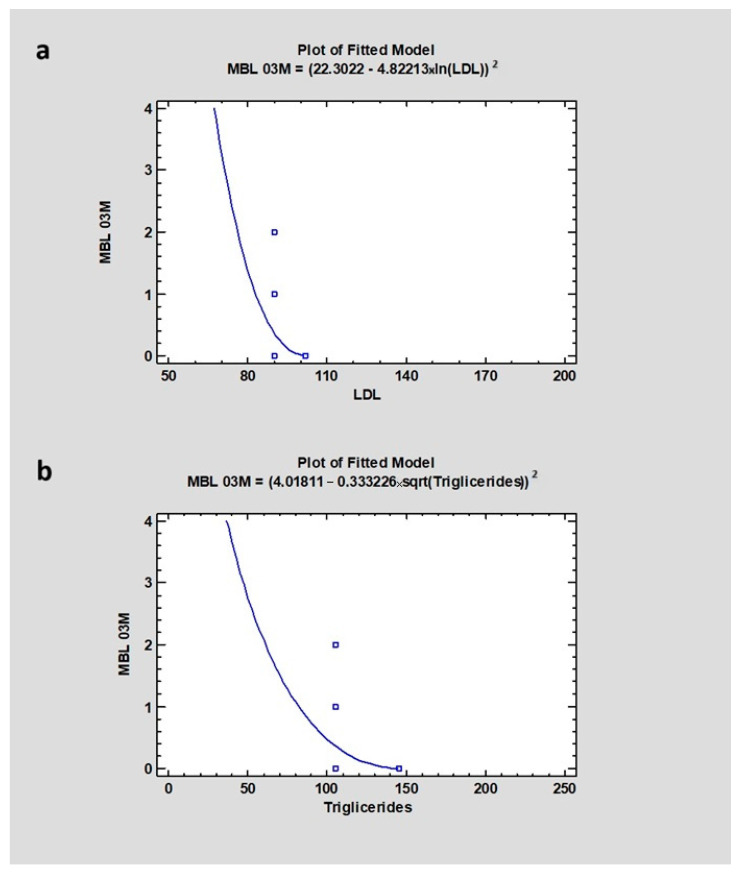
Dependencies for all samples: (**a**) Dependency of LDL on marginal bone loss after 3 months of healing. (**b**) Dependency of triglycerides on marginal bone loss after 3 months of healing. Abbreviations: MBL03—marginal bone loss after 3 months of healing; LDL—low-density lipoproteins.

**Figure 8:**
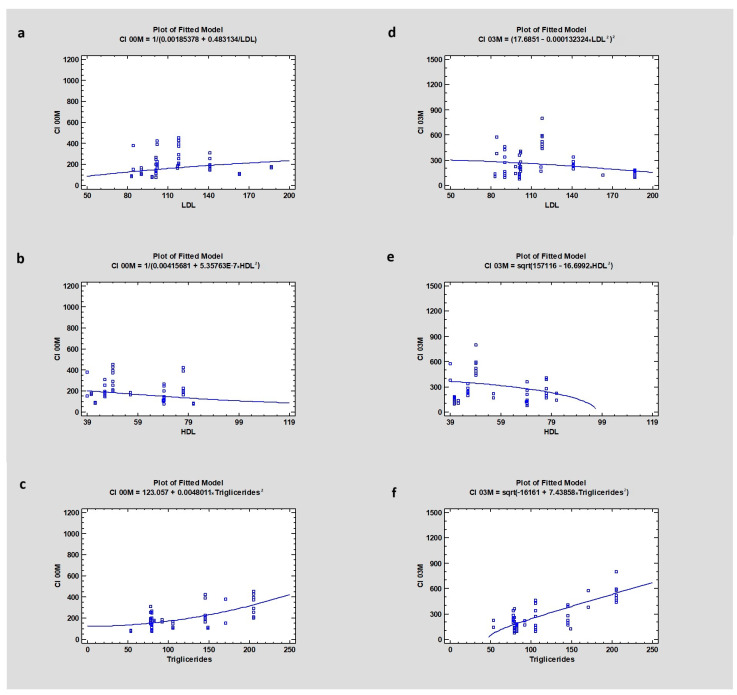
Dependencies for all samples: (**a**) Dependency of LDL on Corticalization Index in the initial period. (**b**) Dependency of HDL on Corticalization Index in the initial period. (**c**) Dependency of Triglycerides on Corticalization Index in the initial period. (**d**) Dependency of LDL on Corticalization Index after 3 months of healing. (**e**) Dependency of HDL on Corticalization Index after 3 months of healing. (**f**) Dependency of Triglycerides on Corticalization Index after 3 months of healing. Abbreviations: CI00M—Corticalization Index in the initial period; CI03M—Corticalization Index after 3 months of healing; LDL—low-density lipoproteins; HDL—high-density lipoproteins.

The research revealed a weak correlation between LDL level (mean 131 ± 34.1) and CI00M (CC = 0.28), CI03M (CC = −0.26), and also MBL after 3 months of healing (CC = −0.6), where the *p*-value in all cases was higher than 0.05, indicating no statistical significance. In the case of HDL, where the mean value was 64 ± 15.9, the study showed a dependency with CI00M (CC = 0.06) and CI03M (CC = −0.22), with a *p*-value higher than 0.05. The level of triglycerides (mean value 94 ± 50.52) in blood serum revealed a correlation with CI00M (CC = 0.62) and with CI after 3 months of healing (CC = 0.78) with *p* = 0, which was statistically significant, and no correlation with marginal bone loss after 3 months (CC = −0.60), with a *p* > 0.05 (Table 3 and Table 4) (Figure 7 and Figure 8).

The study showed a weak correlation between MBL after 3 months and the Corticalization Index on the day of surgery (CC = −0.11; *p* = 0.17) and after 3 months (CC = 0.46; *p* < 0.01).

## 4. Discussion

An uncontrolled growth of BMI may lead to obesity. This condition is commonly known as unhealthy, where adipose tissue has an influence, for example, on estrogen steroid hormone stimulation. Estrogens play a key role in skeletal homeostasis, promoting bone formation, reducing bone resorption (protecting the bone), and acting as antioxidants. The correlation between antioxidants and dental implants made of titanium alloys has also been investigated previously. Higher levels of IL-6 and TNF-α in obesity lead to increased osteoclastogenesis and bone resorption [15,16,17,29,30]. The research noted that different BMI levels may affect greater or smaller marginal bone loss. A higher BMI level may be correlated with smaller MBL and vice versa. The phenomenon of corticalization also changes. A higher BMI status is associated with a lower level of CI on the day of surgery and also after 3 months of healing. In this case, a higher value of CI did not have a positive impact on marginal bone protection, and contrary to the literature, a higher BMI level was correlated with lower atrophy of marginal bone.

Constant or high frequencies/doses of PTH induce the osteoclastogenesis process and impact bone resorption. Lower and rarer doses of PTH have a positive impact on bone remodeling [18,19,31]. This research correlates with other publications. Additionally, it shows again that higher values of CI do not provide bony protection. A growing level of parathyroid hormone may be correlated with a greater atrophy of marginal bone around dental implants. Higher values of CI correlated with higher PTH levels were observed.

The direct impact of TSH on bone metabolism is protective. Indirectly, as mentioned earlier, hypothyroidism leads to obesity (BMI > 30). The issue of BMI was discussed in the first paragraph. Hyperthyroidism indirectly leads to osteoporosis, which is a condition characterized by weak mineralization of the human skeleton [21,32]. This research did not reveal a strong correlation between TSH levels and marginal bone loss or the Corticalization phenomenon. However, it was shown that higher TSH levels correspond to higher CI values after 3 months of healing. The research did not indicate any changing tendencies in any case. Future studies should be conducted separately on groups of men and women.

A greater correlation was observed between phosphates and marginal bone loss compared to the correlation between calcium levels and MBL. Lower values of phosphates in blood serum were correlated with much higher atrophies around dental implants. Additionally, lower values of phosphates were correlated with higher values of the Corticalization Index on the day of surgery and 3 months after the healing process. This suggests that the higher the CI, the higher the MBL after 3 months, indicating that CI does not have a positive impact on marginal bone around implants.

There may be a correlation observed between the level of phosphates in blood serum and PTH levels. This research confirmed that a higher level of phosphates leads to a higher level of PTH and decreased efflux of calcium from bone [26,33]. Calcium levels were correlated with the Corticalization Index—the higher the level of calcium in blood serum, the greater the CI. The research also showed that there may be a suspected correlation between calcium levels and marginal bone loss. A lower level of calcium ions corresponds to a higher level of marginal bone loss; conversely, if the level of calcium ions is closer to normal, the MBL is much lower. According to the available literature and publications, calcium plays a role in the mineralization of bones [34,35]. This research confirmed that the more calcium ions there are, the higher the corticalization of the bone near dental implants.

Vitamin D deficiency may lead to bone loss caused by secondary hyperparathyroidism. A decreased level of this vitamin may also lead to osteoporosis, mineralization defects, and bone fractures. In the long term, it can lead to osteomalacia [36,37,38]. The study confirmed that changes in vitamin D levels may be correlated with bone changes. A decrease in vitamin D was correlated with higher marginal bone loss. The Corticalization Index tends to increase with a higher level of vitamin D (also in the case after 3 months of healing). In this case, it may be suspected that an increasing value of corticalization correlated with a growing level of vitamin D may protect the bone near the dental implants.

Research presents many factors from the general patient’s condition that can change bone structure around the dental implants in the early observation follow-up. It is important to consider that implant failure can occur early or late in the healing/use period. Risk factors and contraindications that can be detected before implantations were many times listed and checked [39,40,41,42]. Further research on the corticalization phenomenon is needed to see how GC may correlate with this indicator at later follow-up periods.

## 5. Conclusions

A correlation between the general condition of patients and the Corticalization Index may be observed. Changes in a patient’s general condition have an impact on bone metabolism around the neck of dental implant. If a patient’s condition is not stable, one should consider postponing dental implant insertion. Many doctors involved in implantology do not order any tests before implantological treatment. Is this correct? In addition, the blood tests presented in the paper should be performed after the implantation through the observation period.

The presented research is another attempt to characterize the Corticalization Index. The study once again shows the two sides of the corticalization phenomenon. It remains heterogeneous and depends on the studied factors. It is not confirmed that the Corticalization Index is an indicator of marginal bone loss around dental implants. The authors again emphasize that while the Corticalization Index as a new phenomenon occurs, further study is still needed.

One limitation of this study was that we did not carry out laboratory tests after 3 months of follow-up. 

## Figures and Tables

**Table 1:** Inclusion criteria for the research [28].

Inclusion Criteria
18 years of age
Bleeding on gingival probing < 20%
Probing depth ≤ 3 mm
Good oral hygiene
Regular follow up’s
2D RTG images taken during the regular check
Laboratory test: PTH, TSH, ions Ca^2+^, HbA1c, vitamin D
Bone densitometry
Smoking

**Table 2:** Exclusion criteria for the research [28].

Exclusion Criteria
Lack of X-rays
Defective X-ray images in the visual assessment
Lack of laboratory tests
Uncontrolled internal co-morbidity: diabetes mellitus, thyroid dishormonoses, rheumatoid disease and other immunodeficiencies
A history of oral radiation therapy
Past or current use of cytostatic drugs
Soft and bone tissue augmentation
Low quality or lack of follow-up radiographs

**Table 3:** This table presents the median values for general factors. Abbreviations: MBL03M—marginal bone loss after 3 months of healing; CI00M—Corticalization Index in the initial period; CI03M—Corticalization Index after 3 months of healing; BMI—Body Mass Index; TSH—thyroid-stimulating hormone, PTH—parathormone; HDL—high-density lipoprotein; LDL—low-density lipoprotein.

	Median Value	Standard Deviation
MBL03M	0 mm	±0.78 mm
CI00M	146.2	±98.02
CI03M	170.6	±112.65
BMI [kg/m^2^]	26.05	±4.26
PTH [pg/mL]	38	±20.05
TSH [mIU/mL]	1.56	±1.62
Calcium level in blood serum	9.5	±2.63
Phosphates level in blood serum	3.3	±7.90
Vit 25(OH)D level in blood serum	23.75	±13.55
LDL	131	±34.09
HDL	64	±15.94
Triglycerides	94	±50.52

**Table 4:** This table presents the *p*-value and the value for the correlation coefficient. Abbreviations: BMI—Body Mass Index; PTH—parathormone; TSH—thyroid-stimulating hormone; LDL—low-density lipoprotein; HDL—high-density lipoprotein.

	Marginal Bone Loss after 3 Months		Corticalization Index in the Day of Surgery		Corticalization Index after 3 Months	
	*p* Value	Correlation Coefficient	*p* Value	Correlation Coefficient	*p* Value	Correlation Coefficient
BMI [kg/m^2^]	0.49	0.09	0.02	−0.11	0.042	−0.10
PTH [pg/mL]	0.18	0.14	0.0043	0.12	0.0022	0.13
TSH [mIU/mL]	0.13	0.16	0.04	−0.08	0.02	0.10
Calcium level in blood serum	0.23	0.13	0.02	0.09	0.31	0.04
Phosphates level in blood serum	0.03	0.24	0.0007	−0.14	0.0000	−0.23
Vit 25(OH)D level in blood serum	0.0004	0.75	0.03	0.29	0.08	−0.25
LDL	0.07	−0.60	0.06	0.28	0.06	−0.26
HDL	-	-	0.06	0.29	0.14	−0.22
Triglycerides	0.07	−0.60	0.00	0.63	0.0000	0.78

## Data Availability

The data on which this study is based will be made available upon request at https://www.researchgate.net/profile/Tomasz-Wach accessed on 1 May 2024.

## References

[1] Wach T., Skorupska M., Trybek G. (2022). Are Torque-Induced Bone Texture Alterations Related to Early Marginal Jawbone Loss?. J. Clin. Med..

[2] Kowalski J., Lapinska B., Nissan J., Lukomska-Szymanska M. (2021). Factors influencing marginal bone loss around dental implants: A narrative review. Coatings.

[3] Jansson L., Lavstedt S. (2002). Influence of smoking on marginal bone loss and tooth loss—A prospective study over 20 years. J. Clin. Periodontol..

[4] Di Domênico M.B., Collares K.F., Bergoli C.D., Dos Santos M.B.F., Corazza P.H., Özcan M. (2021). Factors related to early marginal bone loss in dental implants—A multicentre observational clinical study. Appl. Sci..

[5] Galindo-Moreno P., Catena A., Pérez-Sayáns M., Fernández-Barbero J.E., O’Valle F., Padial-Molina M. (2022). Early marginal bone loss around dental implants to define success in implant dentistry: A retrospective study. Clin. Implant. Dent. Relat. Res..

[6] Gehrke S.A., Júnior J.A., Treichel T.L.E., do Prado T.D., Dedavid B.A., de Aza P.N. (2022). Effects of insertion torque values on the marginal bone loss of dental implants installed in sheep mandibles. Sci. Rep..

[7] Szpak P., Szymanska J. (2018). The relationship between marginal bone loss around dental implants and the specific characteristics of implant-prosthetic treatment. Curr. Issues Pharm. Med. Sci..

[8] Wozniak L., Ratajczak-Wrona W., Borys J., Antonowicz B., Nowak K., Bortnik P., Jablonska E. (2021). Levels of biological markers of nitric oxide in serum of patients with mandible fractures. J. Clin. Med..

[9] Zhang Y., Zhang J., Sun B., Ma L., Ma Y. (2024). Catalpol Promotes Osseointegration of Titanium Implants under Conditions of Type 2 Diabetes via AKT/GSK3β/FYN Pathway-Mediated NRF2 Activation. ACS Omega.

[10] Choukroun E., Parnot M., Surmenian J., Gruber R., Cohen N., Davido N., Simonpieri A., Savoldelli C., Afota F., El Mjabber H. (2024). Bone Formation and Maintenance in Oral Surgery: The Decisive Role of the Immune System—A Narrative Review of Mechanisms and Solutions. Bioengineering.

[11] Parrini S., Chisci G., Leoncini S., Signorini C., Volpi N., Capuano A., Ciccoli L., De Felice C. (2012). F 2-Isoprostanes in soft oral tissues and degree of oral disability after mandibular third molar surgery. Oral Surg. Oral Med. Oral Pathol. Oral Radiol..

[12] Duttenhoefer F., Fuessinger M.A., Beckmann Y., Schmelzeisen R., Groetz K.A., Boeker M. (2019). Dental implants in immunocompromised patients: A systematic review and meta-analysis. Int. J. Implant. Dent..

[13] Fiorillo L., Cicciù M., Tözüm T.F., D’Amico C., Oteri G., Cervino G. (2020). Impact of bisphosphonate drugs on dental implant healing and peri-implant hard and soft tissues: A systematic review. BMC Oral Health.

[14] Baseri M., Radmand F., Hamedi R., Yousefi M., Kafil H.S. (2020). Immunological Aspects of Dental Implant Rejection. BioMed Res. Int..

[15] Cao J.J. (2011). Effects of obesity on bone metabolism. J. Orthop. Surg. Res..

[16] Savvidis C., Tournis S., Dede A.D. (2018). Obesity and bone metabolism. Hormones.

[17] Rinonapoli G., Pace V., Ruggiero C., Ceccarini P., Bisaccia M., Meccariello L., Caraffa A. (2021). Obesity and bone: A complex relationship. Int. J. Mol. Sci..

[18] Chen T., Wang Y., Hao Z., Hu Y., Li J. (2021). Parathyroid hormone and its related peptides in bone metabolism. Biochem. Pharmacol..

[19] Silva B.C., Bilezikian J.P. (2015). Parathyroid hormone: Anabolic and catabolic actions on the skeleton. Curr. Opin. Pharmacol..

[20] Lombardi G., Di Somma C., Rubino M., Faggiano A., Vuolo L., Guerra E., Contaldi P., Savastano S., Colao A. (2011). The roles of parathyroid hormone in bone remodeling: Prospects for novel therapeutics. J. Endocrinol. Investig..

[21] Delitala A.P., Scuteri A., Doria C. (2020). Thyroid Hormone Diseases and Osteoporosis. J. Clin. Med..

[22] Zhu S., Pang Y., Xu J., Chen X., Zhang C., Wu B., Gao J. (2022). Endocrine Regulation on Bone by Thyroid. Front. Endocrinol..

[23] Polska E., Williams G.R. (2009). PRACE POGLĄDOWE/REWIEVS Actions of thyroid hormones in bone Wpływ hormonów tarczycy na tkankę kostną. Pol. J. Endocrinol. Tom.

[24] Galliford T.M., Murphy E., Williams A.J., Bassett J.H.D., Williams G.R. (2005). Effects of thyroid status on bone metabolism: A primary role for thyroid stimulating hormone or thyroid hormone?. Minerva Endocrinol..

[25] Tuchendler D., Bolanowski M. (2014). The influence of thyroid dysfunction on bone metabolism. Thyroid. Res..

[26] Levine B.S., Rodríguez M., Felsenfeld A.J. (2014). Serum calcium and bone: Effect of PTH, phosphate, vitamin D and uremia. Nefrologia.

[27] Yin W., Li Z., Zhang W. (2019). Modulation of bone and marrow niche by cholesterol. Nutrients.

[28] Wach T., Okulski J., Zielí Nski R., Trybek G., Michcik A., Kozakiewicz M. (2024). New Radiological Corticalization Index as an Indicator of Implant Success Rate Depending on Prosthetic Restoration-5 Years of Follow-Up. Diagnostics.

[29] Gkastaris K., Goulis D.G., Potoupnis M., Anastasilakis A.D., Kapetanos G. Obesity, Osteoporosis and Bone Metabolism. http://www.ismni.org.

[30] Zalewska A., Antonowicz B., Szulimowska J., Zieniewska-Siemieńczuk I., Leśniewska B., Borys J., Zięba S., Kostecka-Sochoń P., Żendzian-Piotrowska M., Giudice R.L. (2023). Mitochondrial Redox Balance of Fibroblasts Exposed to Ti-6Al-4V Microplates Subjected to Different Types of Anodizing. Int. J. Mol. Sci..

[31] Kroll M.H., Kroll M.H. (2000). Parathyroid Hormone Temporal Effects on Bone Formation and Resorption. Bull. Math. Biol..

[32] Brancatella A., Marcocci C. (2020). TSH suppressive therapy and bone. Endocr. Connect..

[33] Lederer E. (2014). Regulation of serum phosphate. J. Physiol..

[34] Vannucci L., Fossi C., Quattrini S., Guasti L., Pampaloni B., Gronchi G., Giusti F., Romagnoli C., Cianferotti L., Marcucci G. (2018). Calcium Intake in Bone Health: A Focus on Calcium-Rich Mineral Waters. Nutrients.

[35] Fraser W.D., Colston K.W., Stevenson J.C. (2013). Bone and Calcium Metabolism. The Immunoassay Handbook: Theory and Applications of Ligand Binding, ELISA and Related Techniques.

[36] Lips P., Van Schoor N.M. (2011). The effect of vitamin D on bone and osteoporosis. Best Pract. Res. Clin. Endocrinol. Metab..

[37] Anderson J.J. (1996). Symposium: Nutritional Advances in Human Bone Metabolism. J. Nutr..

[38] Yu F., Laura B., Buzatu R., Buzatu R., Luca M.M. (2024). Impact of Vitamin D on Osseointegration in Dental Implants: A Systematic Review of Human Studies. Nutrients.

[39] Gómez-De Diego R., Del M., Mang-De La Rosa R., Romero-Pérez M.J., Cutando-Soriano A., López-Valverde-Centeno A. (2014). Indications and contraindications of dental implants in medically compromised patients: Update. Med. Oral Patol. Oral Cir. Buccal.

[40] Darby I. (2000). Risk factors for periodontitis & peri-implantitis. Periodontology.

[41] Do T.A., Le H.S., Shen Y.W., Huang H.L., Fuh L.J. (2020). Risk Factors related to Late Failure of Dental Implant-A Systematic Review of Recent Studies. Int. J. Environ. Res. Public Health.

[42] Kullar A.S., Miller C.S. (2019). Are There Contraindications for Placing Dental Implants?. Dent. Clin..

